# Association Between *BMP2* Functional Polymorphisms and Sheep Tail Type

**DOI:** 10.3390/ani10040739

**Published:** 2020-04-23

**Authors:** Zengkui Lu, Jianbin Liu, Jilong Han, Bohui Yang

**Affiliations:** 1 Lanzhou Institute of Husbandry and Pharmaceutical Sciences, Chinese Academy of Agricultural Sciences, Lanzhou 730050, China; luzengkui@caas.cn (Z.L.); liujianbin@caas.cn (J.L.); 15294157518@163.com (J.H.); 2 Sheep Breeding Engineering Technology Research Center of Chinese Academy of Agricultural Sciences, Lanzhou 730050, China

**Keywords:** *BMP2*, sheep, tail type, SNPs, preadipocytes

## Abstract

**Simple Summary:**

We previously detected selection markers of sheep with different tail types and found that bone morphogenetic protein 2 (*BMP2*) was strongly selected. We therefore predicted that *BMP2* might be involved in fat deposition in sheep tails or related to sheep tail type. However, no studies have investigated the molecular mechanism of *BMP2* in the formation of sheep tails. In this study, we performed flight mass spectrometry genotyping on the *BMP2* gene of 533 sheep with different tail types and functionally verified our results at the cellular level. This aimed to provide basic research data for the regulation of fat deposition in sheep tails and to identify genetic markers for molecular breeding.

**Abstract:**

Bone morphogenetic protein 2 (*BMP2*) is strongly selected in both fat-tailed and thin-tailed sheep and may be a candidate gene for sheep tail type selection. However, the mechanism of action of *BMP2* in sheep tail fat deposition remains unclear. This study investigated genetic variation and haplotype combinations of the *BMP2* gene in sheep with different tail types, aiming to reveal the molecular mechanism of *BMP2* in sheep tail fat deposition. We detected a total of three single nucleotide polymorphisms (SNPs) (g.48401619 T > A, g.48401272 C > A, and g.48401136 C > T) among 533 sheep. The alleles and genotype frequencies of these SNPs were in Hardy–Weinberg equilibrium and showed significant correlations with tail length. Linkage disequilibrium existed between the g.48401272 C > A and g.48401136 C > T sites, where CACT was the predominant genotype. At the cellular level, the expression levels of peroxisome proliferator-activated receptor gamma (*PPARγ*) and lipoprotein lipase (*LPL*) were upregulated after *BMP2* overexpression; there were significantly higher levels of *PPARγ* than controls at 0 d and 1 d, and of *LPL* than controls at 1 d and 7 d. These results indicate that the *BMP2* gene may participate in sheep tail fat deposition and could be used for molecular-marker-assisted selection of sheep tail type.

## 1. Introduction

According to the Food and Agriculture Organization of the United Nations (http://www.fao.org/home/en/) statistics, China is the largest producer and consumer of lamb worldwide. Therefore, safeguarding the supply of lamb is of great importance for promoting the development of the national economy. With improvements of human living standards and changes to dietary habits, low-fat lamb products are becoming increasingly popular.

It has been suggested that the world’s first wild sheep were thin-tailed, and that fat-tailed sheep gradually evolved as a result of natural and artificial selection [1]. Fat deposition in a sheep’s tail is an adaptive trait similar to the fat in the hump of a camel [2]. Sheep tail fat provides sufficient energy to survive severe winters with a shortage of forage and is also a food source for people during famine [3,4]. However, as consumption concepts change, fat is becoming a less desirable and less essential trait. Additionally, fat deposits in the tail can reduce those in other parts of the body, which in turn affects the quality of mutton [5]. The preference of both mutton producers and consumers is that the meat can be improved with less fat in sheep tails. Therefore, investigating the molecular regulation mechanisms of fat deposition in sheep tails is of great value to the meat production industry.

Sheep can be divided into short-thin-tailed sheep, long-thin-tailed sheep, short-fat-tailed sheep, long-fat-tailed sheep, and fat-rumped sheep based on the characteristics and size of the tail [6]. It is thought that fat-tailed sheep evolved from thin-tailed sheep because such a biological characteristic is generated to adapt to a specific environment [1]. However, excessive fat deposition in the tails of sheep greatly reduces the financial viability of sheep farming. In terms of reproduction, sheep with small tails are convenient for breeding and improving the pregnancy rate of ewes because hypertrophy of the tail hinders natural mating, requiring additional labor to help breeding. It is reported that the bone morphogenetic protein 2 (*BMP2*) gene is strongly selected in fat-tailed and thin-tailed sheep, and it is predicted that *BMP2* may be related to the sheep tail type and participate in tail fat deposition [7,8], and can be considered a candidate gene for selection for sheep tail type [9,10,11,12].

BMP2 belongs to the transforming growth factor β superfamily. It has extensive biological activity and plays an important role in the processes of skeletal muscle development, osteogenic differentiation, and adipogenic differentiation [13,14,15]. *BMP2* is expressed in human adipose tissues and has the highest expression level in obese individuals [16]. In murine pluripotent mesenchymal cell line C3H10T1/2, *BMP2* activates peroxisome-proliferator-activated receptor gamma (*PPARγ*) to induce adipogenesis through the Smad and p38 mitogen-activated protein kinase (p38MAPK) signal pathways [17]. However, the mechanism by which *BMP2* is involved in fat deposition in sheep tails remains unclear. The aim of this study was to clarify the molecular mechanism of action of *BMP2* in sheep tail fat deposition and provide theoretical support for breeding sheep breeds with specific tail types.

## 2. Materials and Methods

### 2.1. Animals and Measurement of the Tail Type Phenotype

All experimental protocols and procedures were approved by the Institutional Animal Care and Use Committee of Lanzhou Institute of Husbandry and Pharmaceutical Science of Chinese Academy of Agricultural Sciences (Approval No. NKMYD201805; dated: 18 October 2018). Blood was collected from the jugular veins of 533 sheep (24 months old), including 208 Hu sheep (short-fat-tailed sheep, Wuwei, Gansu, China), 171 Tibetan sheep (short-thin-tailed sheep, Tianzhu, Gansu, China), and 154 hybrid sheep (Dorper × Hu, short-fat-tailed sheep, Luoyang, Henan, China). Then, the blood was added to an ethylene diamine tetraacetic acid anticoagulant tube at −20 °C for storage. At the time of sampling, tail length, width, and circumference for each sheep were measured. Tail length was defined as the distance from the leading edge of the first tail vertebra to the tail end, the tail width was the straight line distance from the widest part of the underside of the tail, and tail circumference was the measurement around the widest part of the tail.

### 2.2. PCR Amplification and MassARRAY Genotyping

Genomic DNA was extracted from blood using the TIANamp Genomic DNA Kit (TIANGEN Biotech, Beijing, China) according to the manufacturer’s instructions. The DNA integrity and purity were determined using 1.5% agarose gel electrophoresis and a NanoDrop 2000 spectrophotometer (Thermo, MA, USA). We selected 30 Hu and 30 Tibetan sheep DNA samples at random and diluted them to 50 ng/L. Hu and Tibetan sheep DNA mixing pools were constructed based on 2 µL samples taken from each sample and mixed equally. According to the sheep *BMP2* sequence information in GenBank (XM_004014353.3), 10 pairs of primers were designed for all three exons and 1000 bp upstream and downstream sequences of the *BMP2* gene using Oligo7 software (Supplementary Table S1). The mixed pool of DNA was used for PCR amplification, which consisted of the following: 12.5 µL of 2 × Taq PCR Master Mix, 1 µL of Primer-F, 1 µL of Primer-R, 2 µL of DNA, and 8.5 µL of ddH_2_O. The PCR reaction procedure was as follows: 94 ℃ for 5 min, followed by 35 cycles of 95 ℃ for 30 s, Tm for 30 s, and 72 °C for 30 s, followed by 72 ℃ for 10 min. PCR products were detected by 1.5% agarose gel electrophoresis, cleaned using EasyPure Quick Gel Extraction Kit (TransStart Green, Beijing, China), and submitted to TianYi-HuiYuan biotechnology company (Beijing, China) for Sanger sequencing. The sequencing results were processed using DNAMAN (https://www.lynnon.com) and Chromas 2 software (http://technelysium.com.au/wp/chromas) to perform comparative analyses to determine single nucleotide polymorphism (SNP) sites.

Three SNPs were genotyped (obtained from the PCR of *BMP2*) in 533 sheep using matrix-assisted laser desorption/ionization time-of-flight mass spectrometer (MALDI-TOF-MS). First, based on SNP locus information, single-base amplification and extension primers of the site to be tested were designed using Sequenom Assay Design 3.1 (iPlex assay, Sequenom, San Diego, CA, USA). The PCR amplification system was as follows: 0.65 µL of PCR buffer, 0.35 µL of MgCl2 (Agena, CA, USA), 1 µL of dNTP (Agena), 1 µL of Primer, 0.1 µL of HotstarTaq (5 U/µL, Agena), 1 µL of DNA, and 0.9 µL of ddH_2_O. The PCR reaction procedure was as follows: 94 °C for 15 min, followed by 45 cycles of 94 °C for 20 s, Tm for 30 s, and 72 °C for 60 s, followed by 72 °C for 3 min. Second, the remaining deoxynucleoside triphosphates in PCR products were removed using shrimp alkaline phosphatase enzyme (Agena). The reaction system was as follows: 0.23 µL of 10 × SAP buffer (Agena), 0.4 µL of 10 × SAP enzyme, and 2.1 µL of ddH_2_O. The reaction procedure was as follows: 37 °C for 40 min, followed by 85 °C for 5 min. Third, a single-base extension reaction was performed. The reaction system was as follows: 1 µL of 10 × iPLEX buffer plus (Agena), 0.27 µL of iPLEX terminator (Agena), 1.1 µL of Primer Mix, 0.06 µL of iPLEX enzyme (Agena), and 1.05 µL of ddH_2_O. The PCR reaction procedure was as follows: 94 °C for 30 s, followed by 4 cycles of 94 °C for 5 s, Tm for 5 s, and 80 °C for 5 s, followed by 72 °C for 3 min. Finally, after the sample was purified with clean resin (Sequenom), the purified product was spotted using a Mass ARRAY Nano dispenser, transferred to a Spectro CHIP (Sequenom), and analyzed by MALDI-TOF-MS (Sequenom). The original data and genotyping map were acquired using TYPER 4.0 software (https://www.sequenom.com). Information on the MALDI-TOF-MS genotyping primers is provided in Supplementary Table S2.

### 2.3. Cell Culture and Transfection

The *BMP2*-overexpressed lentivirus was constructed by Genechem gene (Genechem, Shanghai, China), the vector name was GV365, and the element sequence was Ubi-MCS-3FLAG-CMV-EGFP. The overexpression-LV-BMP2 was designed according to BMP2 (XM_004014353). The primer 1 was GAGGATCCCCGGGTACCGGTCGCCACCATGGTGGCCGGGACCCGCTGTC, the primer 2 was TCCTTGTAGTCCATACCACGACACCCACAACCCTCGAC, and the negative control was an empty lentivirus vector. The production and titration of lentiviruses are based on the manufacturer’s protocol. Sheep preadipocytes were isolated from the tail fat of 70-day-old Hu sheep fetuses (*n* = 3) as described by Cai et al. [18]. Briefly, ewes that were 3 months pregnant were selected, the fetuses were isolated after slaughter, adipose tissue was collected, and preadipocytes were isolated using collagenase digestion. The cells were cultured in Nutrient Mixture F12 (DMEM/F12, Gibco, MA, USA) supplemented with 10% fetal bovine serum (Hyclone, MA, USA) and incubated at 37 °C with 5% CO_2_. On the following day, cells were cultured in new fresh medium containing the BMP2-overexpression lentiviral vector (Genechem, Shanghai, China) for 12 h. Preadipocytes were then cultured in new differentiation medium containing 10% FBS, 1 μM dexamethasone (Macklin, Shanghai, China), 0.5 mM isobutylmethylxanthine (Macklin), and 10 mg/mL insulin (Macklin) for 2 days, followed by 10 mg/mL insulin alone for 2 days. Virus without BMP2 overexpression served as a negative control. The date that cells were cultured with differentiation medium was set as the first day (1 d).

### 2.4. RNA Extraction and Quantitative Real-Time PCR

Differential cells at 0, 1, 3, 5, and 7 days were collected for total RNA extraction with TRIzol reagent (Invitrogen, MA, USA). The extracted RNA was reverse-transcribed into cDNA using a cDNA Synthesis Kit (TransStart Green, Beijing, China). TransStart Green qPCR SuperMix (TransStart Green, Beijing, China) and a 480 II LightCycler instrument (Roche, Basel, Switzerland) were employed for qRT-PCR, and each sample was analyzed in triplicate to ensure accuracy. The 2^−ΔΔCt^ method was used to calculate the relative expression of target genes (*BMP2*, *PPARγ*, and *LPL*), and β-actin (*ACTB*) was used as a reference gene [19]. Information on the qRT-PCR primers is provided in Supplementary Table S3.

### 2.5. Oil Red O Staining

Oil Red O dye was made by diluting saturated Oil Red O (0.5%, Biotopped, Beijing, Chain) with distilled water at a ratio of 3:2 and then filtering it. On day 7, differentiated cells (3 × 10^5^) were washed twice with phosphate-buffered saline (PBS), fixed with 4% paraformaldehyde for 20 min, and then rinsed with distilled water. The appropriate amount of Oil Red O dye (0.5 mL, Oil Red O:water = 3:2) was added, and the cells were dyed for approximately 10 min; then, the cells were rinsed with distilled water 2 or 3 times, they were observed under a microscope, and photographs were taken.

### 2.6. Statistical Analysis and Bioinformatics

Microsoft Excel 2013 software (Microsoft Inc, Redmond, WA, USA) was used to calculate the genotype frequency, allele frequency, heterozygosity (*He*), effective allele number (*Ne*), and polymorphic information content (*PIC*) of each site. The Hardy–Weinberg (HW) equilibrium was tested for each site using the chi-squared test. We estimated the linkage disequilibrium (LD) among the SNPs identified in *BMP2* gene in this study using Haploview 4.1 software (https://www.broadinstitute.org/haploview/haploview) and identified the haplotype block. In the process, 95% confidence bounds on D’ were generated. We used the *r^2^* to represent the correlation coefficient between the two loci.

The linear model fitted by the least square method in SAS 9.2 software (SAS Institute Inc., NC, USA) was used to analyze the association of individual SNP genotypes on tail length, width, and circumference. Tukey’s honestly significant difference test was used as a post hoc multiple comparison procedure. The data reported are expressed as least squares mean ± standard error. The models used were the single locus model: Y = μ + G + b + e, where Y is the phenotypic value, µ is the population mean value, G is the genotype effect, b is the breed effect, and e is the random residual. The combined loci model was: Y = μ + G_1_ + G_2_ + G_12_ + b + e, where Y is the phenotypic value, µ is the population mean value, G_1_ is the genotype effect of the g.48401136 C > T locus, G_2_ is the genotype effect of the g.48401272 C > A locus, G_12_ is the interaction between the two sites of g.48401136 C > T and g.48401272 C > A, and e is the random residual. Repeated measures ANOVA was carried out using SPSS software (SPSS, Chicago, IL, USA) for statistical analysis of the qRT-PCR data, and all data reported are expressed as mean ± SE. A *p*-value of < 0.05 was considered to be significant. A *p*-value of < 0.01 was considered to be highly significant.

## 3. Results

### 3.1. Population Genetic Analysis of BMP2 Polymorphism

**Figure 1:**
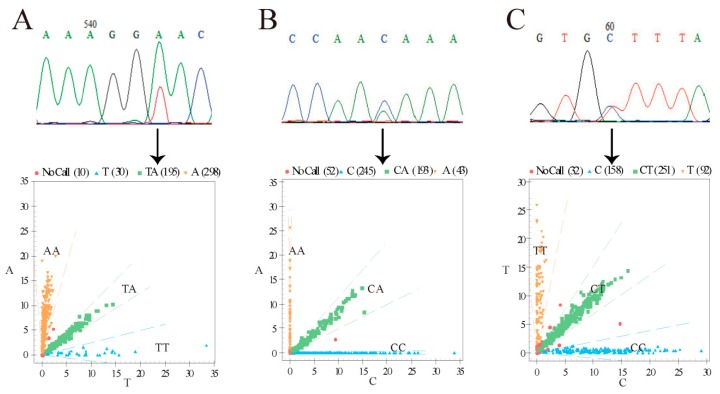
*BMP2* gene pool sequencing and genotyping results. (A) Site of g.48401619 T > A. (B) Site of g.48401272 C > A. (C) Site of g.48401136 C > T. Yellow region, blue region, and green region represent different genotypes. Numbers in brackets indicate number of individuals of the three genotypes. Chromatograms from DNA pool sequencing are shown in the top row, while the mass spectrometry results are presented in the bottom row.

Through pool sequencing, three SNP loci: g.48401619 T > A, g.48401272 C > A, and g.48401136 C > T, were found in the 1000 bp downstream region of the BMP2 gene (Figure 1). These three SNP loci were genotyped by MALDI-TOF-MS (Figure 1). The frequencies and polymorphisms of the three polymorphic loci in three sheep populations are shown in Table 1. g.48401619 T > A had the lowest He in Hu sheep, and g.48401136 C > T had the highest He in Hu sheep. g.48401619 T > A had the lowest Ne in Hu sheep, and g.48401136 C > T had the highest Ne in Hu sheep. Additionally, the Ne of these three loci was close to 2, which indicates moderate polymorphism (0.25 < PIC < 0.5). All three loci were in HW equilibrium (*p* > 0.05).

### 3.2. Association of BMP2 Gene Polymorphism with Tail Phenotypes in Sheep

**Figure 2:**
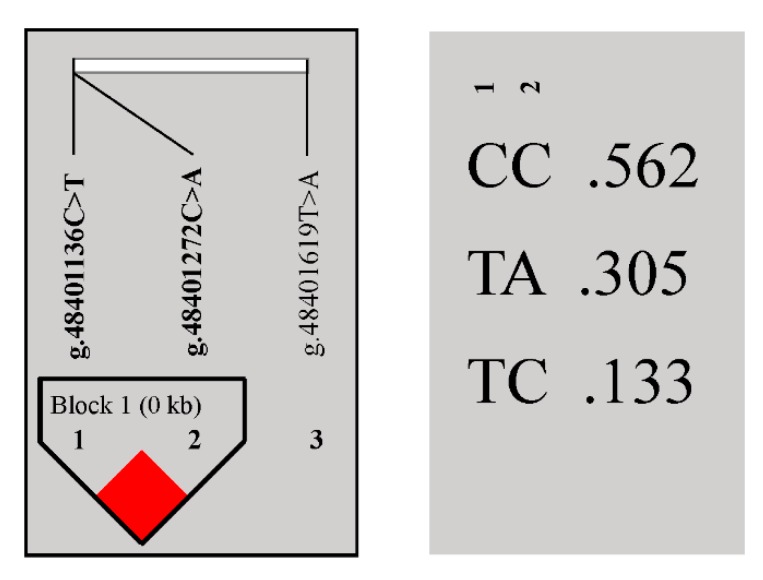
Linkage disequilibrium analysis of the *BMP2* gene.

Statistical Analysis System (SAS) 9.2 software was used to conduct correlation analyses between genotypes and tail data of these three loci, revealing that they were all significantly associated with tail length rather than width or circumference (Table 2). LD analysis showed that g.48401272 C > A and g.48401136 C > T loci were in strong LD (Figure 2, r^2^ = 0.89). Three haplotypes, CC, TA, and TC were detected, of which CC (0.56) was the major haplotype (Figure 2). After combining g.48401272 C > A and g.48401136 C > T, the genotypes AATT, CACT, CATT, CCCC, CCCT, and CCTT were generated, of which CACT (0.35) was the predominant genotype (Supplementary Table S4). Correlation analysis between genotypes and tail data for these six combinations revealed no significant correlation between genotypes (Supplementary Table S4).

### 3.3. Lentiviral Overexpression Efficiency Assay

**Figure 3:**
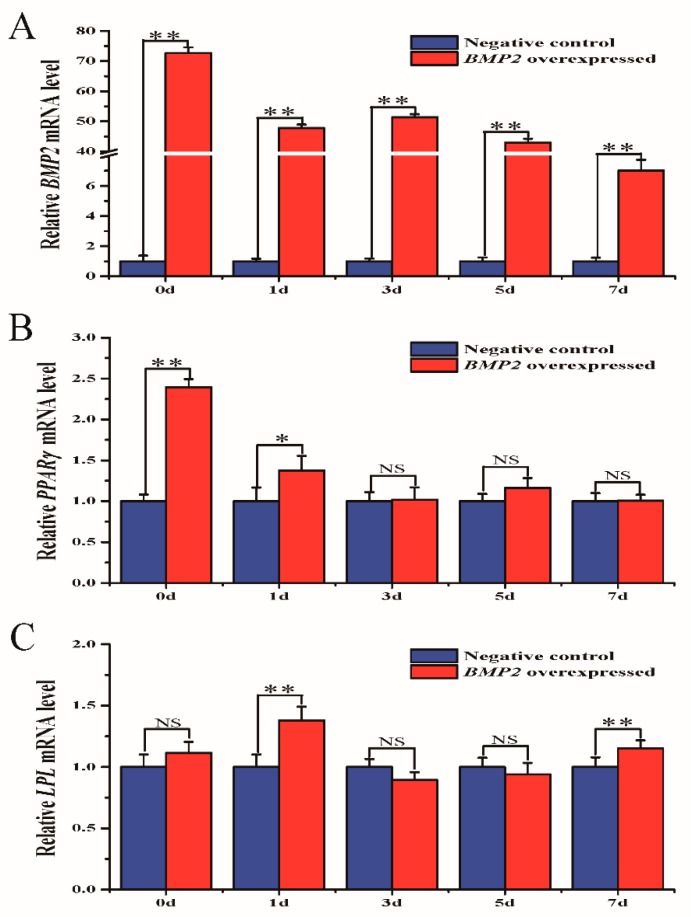
mRNA expression of the *PPARγ* and *LPL* genes. (**A**) mRNA expression of the *BMP2* gene. (**B**) mRNA expression of the *PPARγ* gene. (**C**) mRNA expression of the *LPL* gene. * *p* < 0.05, ** *p* < 0.01, NS: no significant difference. *ACTB* was used as a reference gene.

Sheep preadipocytes were transfected with either BMP2-overexpressed lentivirus or LV-negative control. After transfection for 48 h, it could be observed by microscopy that the cells had a good growth state with clear green fluorescence (Supplementary Figure S1A,B). qRT-PCR on days 0, 1, 3, 5, and 7 after differentiation revealed that *BMP2* expression of the overexpression group was significantly higher than in the control group, thus validating the overexpression effect (Figure 3A).

### 3.4. The Relative Expression of PPARγ and LPL when Overexpressing the BMP2 Gene During Sheep Preadipocyte Differentiation

The influence of overexpressed *BMP2* on *PPARγ* and lipoprotein lipase (*LPL*) gene expression was determined to investigate the effect of the *BMP2* gene on fat deposition. On the day of *BMP2* overexpression and 1 day later, *PPARγ* gene expression in cells in the overexpression group was significantly higher than in the control group (Figure 3B). *LPL* gene expression fluctuated after *BMP2* was overexpressed but was clearly higher in cells in the overexpression group than in the control group on days 1 and 7 (Figure 3C).

### 3.5. Oil Red O Staining

**Figure 4:**
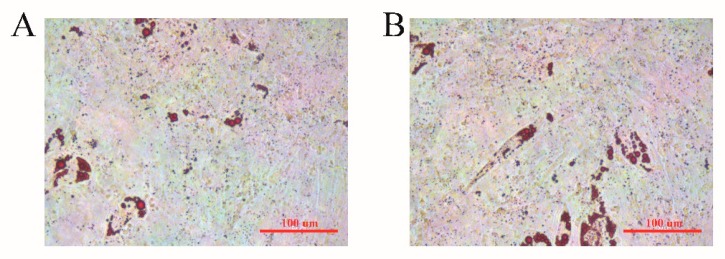
Oil Red O staining when overexpressing the *BMP2* gene in sheep preadipocytes after 7 days of induced differentiation. (A) Control group. (B) *BMP2* overexpressed group.

Sheep preadipocytes were selected on day 7 after induced differentiation to conduct Oil Red O staining. More small oil droplets and fewer large oil droplets were seen in both the overexpression and control *BMP2* groups, and there was no significant difference between groups (Figure 4).

## 4. Discussion

*He*, *Ne*, and *PIC* are often used to measure the degree of genetic variation in a population [20,21] with a higher numerical value representing a greater degree of genetic variation. We found that the g.48401619 T > A site was moderately polymorphic, with lower heterozygosity and a low degree of genetic variation compared with other sites, which may be related to the high degree of selection in the breeding of the experimental population. g.48401272 C > A and g.48401136 C > T sites were moderately polymorphic, with relatively high heterozygosity and greater genetic variation, which may allow more selection effects. The closer the effective number of alleles is to two, the more evenly distributed the site is in the population. The effective number of alleles at the g.48401619 T > A site was lower than that of other sites; however the locus was in HW equilibrium, indicating that g.48401619 T > A is not evenly distributed in the experimental group. This may be related to the environment, selection, or matching system of the experimental group. However, not all groups were continuously under intense selection, as the other sites (g.48401272 C > A and g.48401136 C > T) showed an even distribution in the genome and were in HW equilibrium.

We initially searched for SNP loci in Hu, Tibetan, and hybrid (Dorper × Hu) sheep, but only found some individual mutation loci, which were not significantly associated with tail type data in the corresponding breeds. With increasing age, the tail length, width, and circumference of adult sheep will not increase significantly, and the difference within the same breed is small. Therefore, we finally merged the three breeds of sheep into one large group for correlation analysis. Three mutated sites were identified downstream of the *BMP2* gene, and the polymorphism of the three sites was significantly associated with sheep tail length. Similarly, an SNP site in the 3′ UTR of the human *BMP2* gene is associated with the formation of human subcutaneous fat [22]. It is widely believed that 3′ UTR regulatory regions are involved in post-transcriptional regulation and play an important regulatory role in mRNA stability, gene transcription efficiency, and expression localization [23,24,25]. It remains to be seen whether the three sites we found in the 3′ UTR of the sheep *BMP2* gene contribute to post-transcriptional regulation. Single sites can affect phenotypes; moreover, the linkage of two or more sites may have a greater effect on phenotypes [26]. After the combination of ≥ 2 sites, our analysis was able to take into account the effects and LD of non-allelic genes [27]. Therefore, we conducted LD analysis for these three sites. Linkage disequilibrium existed between g.48401272 C > A and g.48401136 C > T sites, and so the predominant CCCT (14.63%) genotype had a smaller proportion in the population, whereas the inferior CCCC (31.52%) genotype had a larger proportion in the population. This suggests that the selection and retention of CACT (34.71%) genotype individuals could be increased in future breeding processes, while CCCC genotype individuals could be eliminated when appropriate.

To further understand the molecular mechanisms of the *BMP2* gene in sheep fat deposition, we used an exogenously synthesized *BMP2* overexpression lentiviral vector to transfect sheep preadipocytes. After 48 h of transfection, adipogenic differentiation was induced, the expression of marker genes related to adipose differentiation was detected, and Oil Red O staining was conducted. *PPARγ* and *LPL* are both crucial transcriptional regulatory factors that play important regulatory roles in adipocyte differentiation [28,29]. On day 1 of differentiation, *PPARγ* expression increased rapidly and was maintained at a high level on days 3 and 5, which indicates that the cells had entered the differentiation stage. *LPL* expression dropped after peaking on day 5. This is consistent with the finding that *LPL* is expressed at the early stage of fat differentiation [30]. The results of the Oil Red O staining showed that there were no significant differences in lipid droplet accumulation between control and experimental groups, which may be related to the concentration of the added *BMP2* lentivirus or the time of the Oil Red O staining. It has been shown that *PPARγ* expression first increases and then decreases with age in Tan sheep, which is consistent with the results of this study [31]. Similarly, after *BMP2* induction of mouse pluripotent stem cells, Oil Red O staining revealed significant adipogenic differentiation, and the *PPARγ* expression was also upregulated [32]. *LPL* expression in the adipose tissue of sheep was shown by Bonnet et al. to be high and not affected by nutrition level [33]. We found that *LPL* gene expression fluctuated at different differentiation stages; hence, the gene could not be used as a marker gene for fat deposition in the sheep tail.

BMP2 is involved in the bone morphology and body shape development of sheep [34]. In the African clawed frog (*Xenopus laevis*), BMP is closely related to tail development and regeneration, in that the inhibition of *BMP2* expression inhibits tail regeneration [35]. Vertnin (*VRTN*) was reported to be a key candidate gene controlling the number of vertebrae in sheep and pigs [36,37,38]. As a transcriptional inhibitor, *VRTN* independently regulates expression of the *BMP2B* gene in the dorsoventral axis through a combination of its regulatory sequences, thereby enabling embryos to develop normally along the dorsoventral axis [39]. All these results indicate that *BMP2* is directly or indirectly involved in the bone development of animals. *BMP2* promotes both adipogenic differentiation and osteogenic differentiation [16,40]. Compared with short-tailed sheep, long-tailed sheep have more caudal vertebrae and accumulate more fat. Taken together with our finding that three *BMP2* SNP sites were significantly correlated with the tail length of sheep, therefore, it is unknown whether *BMP2* is directly involved in sheep tail fat deposition or indirectly affects the amount of fat deposition by regulating the number of sheep caudal vertebrae (whereby a larger number of caudal vertebrae means more fat deposition); its mechanism of action requires further study.

## 5. Conclusions

In conclusion, the *BMP2* gene is polymorphic in sheep. The g.48401619 T > A, g.48401272 C > A, and g.48401136 C > T loci all show significant association with sheep tail length. Additionally, overexpression of *BMP2* in sheep preadipocytes can promote adipogenic differentiation. This result will be of use in molecular-marker-assisted selection of sheep tail length.

## Figures and Tables

**Table 1:** Diversity parameters for the sheep *BMP2* gene.

Locus	Breed	Genotype	Genotype Frequency	Allele Frequency	*He*	*Ne*	*PIC*	HW Test (*p*-Value)
g.48401619 T > A	Hu sheep	TT (7)	0.03	T (0.19) A (0.81)	0.31	1.45	0.26	0.76
		TA (66)	0.32					
		AA (135)	0.65					
	Tibetan sheep	TT (9)	0.05	T (0.23) A (0.77)	0.35	1.54	0.29	0.88
		TA (59)	0.35					
		AA (103)	0.60					
	Hybrid sheep	TT (12)	0.08	T (0.33) A (0.67)	0.44	1.80	0.34	0.08
		TA (78)	0.50					
		AA (64)	0.42					
g.48401272 C > A	Hu sheep	AA (14)	0.07	A (0.25) C (0.75)	0.37	1.59	0.30	0.64
		CA (75)	0.36					
		CC (119)	0.57					
	Tibetan sheep	AA (24)	0.14	A (0.34) C (0.66)	0.45	1.81	0.35	0.11
		CA (67)	0.39					
		CC (80)	0.47					
	Hybrid sheep	AA (16)	0.10	A (0.35) C (0.65)	0.45	1.83	0.35	0.36
		CA (75)	0.49					
		CC (63)	0.41					
g.48401136 C > T	Hu sheep	CC (58)	0.28	C (0.52) T (0.48)	0.50	2.00	0.37	0.70
		CT (101)	0.48					
		TT (49)	0.24					
	Tibetan sheep	CC (63)	0.37	C (0.59) T (0.41)	0.48	1.93	0.37	0.38
		CT (77)	0.45					
		TT (31)	0.18					
	Hybrid sheep	CC (47)	0.31	C (0.58) T (0.42)	0.49	1.95	0.37	0.10
		CT (85)	0.55					
		TT (22)	0.14					

**Table 2:** Association of different SNP genotypes with tail type traits in sheep.

Locus	Genotype	Tail Length (cm)	Tail Width (cm)	Tail Circumference (cm)
g.48401619 T > A	TT	19.39 ± 0.51 ^ab^	9.85 ± 0.12 ^a^	20.26 ± 0.24 ^a^
	TA	19.24 ± 0.19 ^b^	9.46 ± 0.14 ^a^	19.78 ± 0.28 ^a^
	AA	19.85 ± 0.16 ^a^	9.71 ± 0.37 ^a^	20.06 ± 0.76 ^a^
g.48401272 C > A	AA	19.71 ± 0.36 ^ab^	9.71 ± 0.27 ^a^	20.04 ± 0.55 ^a^
	CA	19.22 ± 0.18 ^b^	9.62 ± 0.13 ^a^	20.09 ± 0.27 ^a^
	CC	19.87 ± 0.178 ^a^	9.75 ± 0.12 ^a^	20.04 ± 0.25 ^a^
g.48401136 C > T	CC	20.02 ± 0.21 ^a^	9.80 ± 0.15 ^a^	20.26 ± 0.31 ^a^
	CT	19.41 ± 0.17 ^ab^	9.54 ± 0.12 ^a^	19.83 ± 0.25 ^a^
	TT	19.33 ± 0.27 ^b^	9.90 ± 0.20 ^a^	20.37 ± 0.40 ^a^

In the same column, values with different lower-case superscripts are significantly different (*p* < 0.05). Data expressed as LSM ± SE.

## Supplementary Materials

The following are available online at https://www.mdpi.com/2076-2615/10/4/739/s1; Table S1: Primers used in this study for PCR, Table S2: Primers used in this study for MALDI-TOF-MS genotyping. Table S3: Primers used in this study for qRT-PCR, Table S4: Associations of haplotype combinations with tail type traits in sheep, Figure S1: Detection of overexpression efficiency of the *BMP2* gene. (A) Positive cells observed using a light microscope (20×). (B) Positive cells observed using a fluorescence microscope (20×).

## Abbreviations

The following abbreviations are used in this manuscript:

ACTB	β-actin;
BMP2	bone morphogenetic protein 2;
He	heterozygosity;
LPL	lipoproteinlipase;
MALDI-TOF-MS	matrix-assisted laser desorption/ionization time-of-flight mass spectrometer;
Ne	effective allele number;
PIC	polymorphic information content;
PPAR*γ*	peroxisome proliferator-activated receptor gamma;
qRT-PCR	quantitative real-time PCR;
SNP	single nucleotide polymorphism;
TGF-β	transforming growth factor β;
VRTN	vertnin.
